# Zero discrimination in practice: resisting anti‐trans backlash in the global HIV response

**DOI:** 10.1002/jia2.70083

**Published:** 2026-02-26

**Authors:** Tonia C. Poteat, L. Leigh Anne van der Merwe, Laylla Monteiro, Sari L. Reisner

**Affiliations:** ^1^ Division of Healthcare in Adult Populations Duke University School of Nursing Durham North Carolina USA; ^2^ Social, Health and Empowerment (S.H.E.) Feminist Collective of Transgender Women of Africa East London South Africa; ^3^ Evandro Chagas National Institute of Infectious Diseases (INI), Oswaldo Cruz Foundation (FIOCRUZ) Rio de Janeiro Brazil; ^4^ Department of Epidemiology University of Michigan School of Public Health Ann Arbor Michigan USA

1

Since its launch by UNAIDS in 2014, Zero Discrimination Day has been observed every March around the world [1]. This observance calls for the eradication of discrimination in all forms and affirms the right of every person to live with dignity—without fear of violence and free from internalized, interpersonal, social and structural stigma. The goal of zero discrimination responds to mounting evidence that punitive laws and abrogation of human rights are not only patently unacceptable but are also significant barriers to engagement in HIV prevention, care and treatment [2]. Achieving this goal will require removing harmful laws, ensuring legal protections and supporting the empowerment of communities most affected by HIV. The HIV community can be a powerful catalyst for change, with a long history of activism and advocacy that have shaped medical, legal and social landscapes of the global HIV response [3].

The 2024 UNAIDS report found that, globally, transgender women bear a 20‐fold higher burden of HIV than the general adult population [4]. While data regarding transgender men are limited, existing evidence also reflects their seven‐fold greater burden of HIV [5]. Data on HIV prevalence among gender nonbinary individuals are scant; however, a recent study across 20 European countries found a self‐reported HIV prevalence of 5.6%, also much higher than their general populations [6]. Despite elevated HIV prevalence, access to and uptake of biomedical HIV services are low [5]. These inequities are driven by stigma, discrimination and social exclusion, and intensified by growing legal, policy, rhetorical and physical attacks across the world that threaten hard‐won gains in the global HIV response. In 2025, UNAIDS warned of the most serious setback in decades due to funding disruptions and deteriorating human rights conditions, including the first rise in countries criminalizing gender diversity and same‐sex relationships since 2008 [7]. These developments directly impede access to HIV services, particularly for transgender communities already experiencing disproportionate HIV burden and structural barriers to prevention and care.

Across Europe and Central Asia, Transgender Europe's 2025 Trans Rights Index documented an unprecedented reversal: setbacks in transgender rights now outweigh progress, with bans or restrictions on legal gender recognition and diluted antidiscrimination protections in multiple countries [8]. While some advances occurred (e.g. Germany's self‐determination law [9]), the aggregate trajectory is regressive and tied to broader democratic erosion.

In the United States, a record volume of anti‐transgender bills are targeting healthcare, education, civil rights and daily public participation. By late 2025, more than 1000 bills had been introduced across 49 of 50 states, with over 100 enacted. These policies increasingly restrict gender‐affirming care for youth and, in some places, adults, with cascading harms to health and safety [10]. Beyond the United States, the Trump administration's executive orders have emboldened negative ideological stances of African states that criminalize transgender identities [11]. Funding for transgender communities is adversely affected by the paternalistic aid dynamics with developing nations, where financial stipulations emphasize American ideals and undermine African public health priorities, especially relating to key populations [12, 13].

In Brazil, although the country guarantees access to healthcare and legal recognition of gender identity, these gains coexist with persistently high levels of stigma, structural transphobia, social exclusion and premature mortality among transgender people, particularly Black, Indigenous and other racialized transgender women [14]. Brazil remains one of the deadliest countries for transgender people, and HIV prevalence among transgender communities continues to be disproportionately high [5, 15]. While Brazil's health system constitutes one of the largest publicly funded HIV programmes worldwide, transgender people continue to experience systematic discrimination within health services, which contribute to HIV inequities [2]. The Brazilian experience underscores that legal recognition alone—when not accompanied by sustained structural investment, comprehensive anti‐discrimination policies and social protections—is insufficient to reduce inequities and achieve global HIV targets.

In several Latin American countries, including Venezuela, gaps in legal gender recognition combined with migration and forced displacement create structural barriers to HIV services access and achievement of viral suppression [16]. These legal and social constraints compound persistent disparities observed among transgender women in the region. In Asia and the Pacific, restrictive legal environments and intersectional stigma in healthcare obstruct progress in the HIV response [17], including uptake of biomedical prevention [18], despite calls to address health and human rights and community‐engaged implementation strategies for transgender people [19].

Key population programming has already been disrupted by funding shocks; rights rollbacks compound these disruptions, undercutting community‐led services and discouraging health service seeking. UNAIDS warns that failure to restore prevention services could yield 3.3 million additional HIV acquisitions between 2025 and 2030 [7]. Transgender communities, given their disproportionate burden and lower service coverage, stand at the acute intersection of these risks. Such hostile legal climates reduce prevention and care engagement [20], while criminalization and stigma impede HIV targets and violate obligations under international laws [21]. HIV science is clear: removing punitive laws, protecting gender diversity and investing in community‐led services are necessary to end HIV. Table 1 provides our recommendations for trans‐inclusive global objectives to advance the goals of zero discrimination.

**Table 1:** Trans‐inclusive global objectives to advance zero discrimination

Objectives	Actions
1 Protect and expand access to gender‐affirming care	Ensure access to this evidence‐based, lifesaving healthcare, integrating harm reduction strategies where availability is threatened.
2 Safeguard diversity, equity and inclusion programmes	Ensure that gender education, sensitivity trainings and accountability mechanisms persist even under political pressure.
3 Invest in community‐led HIV services	Provide resources for community‐based, trans‐led, peer navigation, prevention programmes and psychosocial support.
4 Advance legal reform	Not only, decriminalize HIV, gender expression and consensual same‐sex sexuality, but also to respect and protect the rights of gender‐diverse people.
5 Protect data, research and education about transgender health	Retain access to existing data sources and fund new studies that address drivers of transgender health inequities. Resist political interference in evidence‐based research, education and care.
6 Align financing with rights and community‐derived priorities	Governments and donors should step into funding gaps to stabilize HIV prevention and care budgets, prioritizing key populations and protecting human rights that enable uptake.
7 Counter disinformation	Opponents of transgender rights often mobilize disinformation to generate anti‐transgender animus. Countering these efforts will require not only rebutting falsehoods but actively shifting and creating new narratives.
8 Identify new allies and deepen existing collaborations	Seek new partnerships with organizations, networks and individuals who have untapped potential to support transgender health and human rights. Maintain and fortify local, regional and global connections with current allies to build stronger, intersectoral coalitions that collectively advance zero discrimination and the rights of transgender people.

Throughout history, every major advance in HIV prevention and care has depended not only on resources and policies, but also on the HIV community's ability to translate its collective power into action. While these goals may seem aspirational, the roadmap for achieving them is clear. This moment demands that the HIV community understands that we have real power and translate that power into practice—deliberatively and courageously using our power to resist erasure of transgender lives. Zero Discrimination Day is not merely commemorative; it is a call to action. As anti‑transgender backlash spreads, the HIV movement must reaffirm that rights are essential for health—without protection of transgender people's dignity, bodily autonomy and access to equitable systems, HIV goals will remain out of reach. We must take pragmatic steps that uphold science and save lives. To end the AIDS pandemic, we must end discrimination.

## COMPETING INTERESTS

All authors declare no competing interests.

## AUTHORS’ CONTRIBUTIONS

TCP created the original draft of the manuscript. SLR, LLAvM and LM provided substantive edits.

## Data Availability

The data that support the findings of this study are available on request from the corresponding author. The data are not publicly available due to privacy or ethical restrictions.

## References

[1] UNAIDS . Zero discrimination day is observed each year on 1 March [Internet]. [cited 2026 Jan 4]. Available from: https://www.unaids.org/en/zero‐discrimination‐day.

[2] Doyle CM , Kuchukhidze S , Stannah J , Flores Anato JL , Xia Y , Logi C , et al. The impact of HIV stigma and discrimination on HIV testing, antiretroviral treatment, and viral suppression in Africa: a pooled analysis of population‐based surveys. SSRN [Internet]. [cited 2026 Jan 4]. Available from: https://ssrn.com/abstract=5225713.10.1016/S2352-3018(25)00325-X41785886

[3] Mbali M . The Treatment Action Campaign and the history of rights‐based, patient‐driven HIV/AIDS activism in South Africa. In: Jones P and Stokke K , editors. Democratising development. Brill Nijhoff Law Specials, Volume: 64. Leiden, The Netherlands; 2005. p.213–243. 10.1163/9789047415732_011.

[4] Joint United Nations Programme on HIV/AIDS (UNAIDS) . HIV and transgender people: thematic briefing note—2024 global AIDS update. Geneva: UNAIDS; 2024 [cited 2026 Jan 4]. Available from: https://www.unaids.org/sites/default/files/media_asset/2024‐unaids‐global‐aids‐update‐transgender‐people_en.pdf.

[5] Stutterheim SE , van Dijk M , Wang H , Jonas KJ . The worldwide burden of HIV in transgender individuals: an updated systematic review and meta‐analysis. PLoS One. 2021;16(12):e0260063. 34851961 10.1371/journal.pone.0260063 PMC8635361

[6] Wang H , Kolstee J , Casalini JL , Hakim S , Zimmermann HM , Jonas KJ . Likelihood of HIV and recent bacterial sexually transmitted infections among transgender and non‐binary individuals in 20 European countries, October 2023 to April 2024. Eurosurveillance. 2024;29(48):2400347. doi: 10.2807/1560-7917.ES.2024.29.48.2400347. 39611207 PMC11605802

[7] United Nations . Global HIV response facing worst setback in decades. UNAIDS warns. UN News; 2025 [cited 2026 Jan 4]. Available from: https://news.un.org/en/story/2025/11/1166449.

[8] Transgender Europe (TGEU) . Trans Rights Index & Map 2025: the new trans tipping point and Europe's struggle for self‐determination. 2025 [cited 2026 Jan 4]. Available from: https://tgeu.org/trans‐rights‐index‐map‐2025.

[9] German missions in the United States. Self‐determination. 2024 [cited 2026 January 19]. Available from: https://www.germany.info/us‐en/service/04‐familymatters/self‐determination‐2671874.

[10] Trans Legislation Tracker . Learn: U.S. anti‐trans legislation history. [cited 2026 Jan 4]. Available from: https://translegislation.com/learn.

[11] Shaw A . Impact of executive order pausing U.S. foreign aid on LGBTQI+ people. Los Angeles, CA: Williams Institute; 2025. Available from: https://williamsinstitute.law.ucla.edu/publications/foreign‐aid‐eo‐impact.

[12] Sonjani B , Makitla M . Reflections from the Global South: how the Trump administration impacts trans & intersex people. Iranti; 2025 [cited 2026 Jan 4]. Available from: https://www.iranti.org.za/trump‐administration‐impacts‐trans‐intersex‐people.

[13] Musella AM , Alberti Corseri L . The paternalistic assumptions in the narrative of international aid: an historical overview. Int J Bus Soc Sci. 2021;12(4):9–19. Available from: https://ijbss.thebrpi.org/journals/Vol_12_No_4_April_2021/2.pdf.

[14] Pinheiro TF , de Carvalho PGC , Nolasco G , dos Santos LA , Veras MASM . Difficulties and advances in access to and use of health services by transgender women and travestis in Brazil. Rev Bras Epidemiol. 2024;27(Suppl 1):e240007. Available from: https://www.scielosp.org/article/rbepid/2024.v27suppl1/e240007.supl.1/ 39166579 10.1590/1980-549720240007.supl.1 PMC11338531

[15] Transgender Europe (TGEU) . Will the cycle of violence ever end? TGEU's Trans Murder Monitoring project crosses 5,000 cases. 2024 [cited 2026 Jan 4]. Available from: https://tgeu.org/will‐the‐cycle‐of‐violence‐ever‐end‐tgeus‐trans‐murder‐monitoring‐project‐crosses‐5000‐cases/

[16] Wirtz AL , Guillén JR , Stevenson M , Ortiz J , MÁB T , Page KR , et al. HIV infection and engagement in the care continuum among migrants and refugees from Venezuela in Colombia: a cross‐sectional, biobehavioural survey. Lancet HIV. 2023;10(7):e461–e471. doi: 10.1016/S2352-3018(23)00085-1. 37302399 PMC10336726

[17] Janamnuaysook R , Rosadiño D , Castellanos E . Breaking barriers: addressing transphobia and advancing transgender rights in the Asia‐Pacific and beyond. J Int AIDS Soc. 2024;27(5):e26273. doi: 10.1002/jia2.26273. 38752661 PMC11097620

[18] Janamnuaysook R , Guo Y , Yu YJ , Phanuphak N , Kawichai S , MacDonell K , et al. Lived experiences with pre‐exposure prophylaxis uptake and adherence among transgender women in Thailand: a qualitative study. Sex Health. 2024;21:SH23102. doi: 10.1071/SH23102. 38219741 PMC11536311

[19] Restar A , Minalga BJ , Quilantang MI , Adamson T , Dusic E , van der Merwe LA , et al. Mapping community‐engaged implementation strategies with transgender scientists, stakeholders, and trans‐led community organizations. Curr HIV/AIDS Rep. 2023;20(3):160–169. doi: 10.1007/s11904-023-00656-y. 37012537 PMC10071255

[20] Sullivan P , Brixner D , Lam JT , Hsiao A . Overcoming barriers to HIV prevention: population health considerations on optimizing PrEP access. Am J Manag Care. 2024;30(suppl 11):S207S215. Available from: https://www.ajmc.com/view/overcoming‐barriers‐to‐hiv‐prevention‐population‐health‐considerations‐on‐optimizing‐prep‐access. 10.37765/ajmc.2024.89654 39652788

[21] Office of the United Nations High Commissioner for Human Rights (OHCHR) . HIV/AIDS and human rights. Geneva: OHCHR. [cited 2026 Jan 4]. Available from: https://www.ohchr.org/en/health/hivaids‐and‐human‐rights.

